# Prevalence and Clinical Impact of Human Pegivirus-1 Infection in HIV-1-Infected Individuals in Yunnan, China

**DOI:** 10.3390/v9020028

**Published:** 2017-02-15

**Authors:** Zhijiang Miao, Li Gao, Yindi Song, Ming Yang, Mi Zhang, Jincheng Lou, Yue Zhao, Xicheng Wang, Yue Feng, Xingqi Dong, Xueshan Xia

**Affiliations:** 1 Faculty of Life Science and Technology, Kunming University of Science and Technology, Kunming 650500, China; miaozhijiang@yeah.net (Z.M.); yindisong@163.com (Y.S.); qjyangming@163.com (M.Y.); zy19860908@yeah.net (Y.Z.); 2 Department of Infectious Diseases, Yunnan Provincial Hospital of Infectious Diseases, Kunming 650301, China; gaoli296@aliyun.com (L.G.); zm050306@sohu.com (M.Z.); ljc666yn@163.com (J.L.); wxch62597@foxmail.com (X.W.)

**Keywords:** HPgV-1, HIV-1, HCV, co-infection, injecting drug users, heterosexuals, genotypic diversity, clinical effect

## Abstract

Human Pegivirus-1 (HPgV-1) may have a beneficial impact on disease progression in human immunodeficiency virus-1 (HIV-1) infection. However, analysis of the genotypic diversity of HPgV-1 and its relevance to the progression of HIV-1 disease remains limited. A total of 1062 HIV-1-infected individuals were recruited in all sixteen prefectures of Yunnan province, China. The reverse transcription nested polymerase chain reaction (RT-nPCR), phylogenetic analyses, and clinical data analyses were used to detect HPgV-1 infection, determine genotype, and analyze HPgV-1 genotype impact on HIV-1 disease progression. The overall positive rate of HPgV-1 RNA was 23.4% (248/1062), and the frequency of HPgV-1 infection in injecting drug users (IDUs) (28.5%, 131/460) was significantly higher than in heterosexuals (19.4%, 117/602). Multiple genotypes were identified in 212 subjects with successful sequencing for the *E2* gene, including genotype 7 (55.7%), genotype 3 (34.9%), genotype 4 (4.7%), genotype 2 (3.3%), and an unclassified group (1.4%). Moreover, genotype 7 predominated in IDUs, whereas genotype 3 was the most common in heterosexuals. Our results revealed that HPgV-1 genotype 7 groups exhibited significantly lower HIV-1 viral load and higher CD4^+^ cell counts. This finding suggests that HPgV-1 genotype 7 may be associated with a better progression of HIV-1 disease.

## 1. Introduction

Human pegivirus-1 (HPgV-1), also known as GB virus C (GBV-C) or hepatitis G virus (HGV), is a positive-sense single-stranded RNA (ssRNA) virus that has recently been classified under the *Pegivirus* (pe, persistent; g, GB or G) genus of the *Flaviviridae* family [1,2,3,4]. HPgV-1 possesses a genome of approximately 9.4 kb nucleotides in size that encodes a polyprotein of 2900 amino acids with characteristic structural proteins (E1 and E2) and non-structural motifs (NS2, NS3, NS4A, NS4B, NS5A, and NS5B) which is organized similarly to the genome of the hepatitis C virus (HCV) [4,5]. To date, seven HPgV-1 genotypes have been identified by phylogenetic analysis of the full-length and partial regions of the genome 5′ untranslated region (5’-UTR) and envelope protein 2 (*E2*) [6]. These genotypes display a specific geographical distribution; for instance, the HPgV-1 genotype 1 is predominant in West African, genotype 2 in the United States and Europe, genotype 3 in East Asia, genotype 4 in Myanmar and Vietnam, genotype 5 in South Africa, genotype 6 in Indonesia. More recently, genotype 7 has been found in Yunnan, China [7].

HPgV-1 is a nonpathogenic human virus that is transmitted efficiently via parenteral, vertical, and sexual routes [4,8,9]. As a result of the shared modes of transmission with the human immunodeficiency virus (HIV) and HCV, HPgV-1 infection is more prevalent among HIV-1- and/or HCV-positive individuals. It has been reported that up to 40% of HCV- and/or HIV-1-infected patients are HPgV-1 positive [10,11]. Consistent with these findings, our previous study revealed that 35.8% of HIV-1-infected injecting drug users (IDUs) in Yunnan, China were positive for HPgV-1 RNA [7].

Notably, several studies have confirmed that HPgV-1 does not cause any liver related disease in humans [4,8,12]. However, co-infection with HIV-1 may result in favorable outcomes such as lower mortality rate, slower disease progression, and longer survival [7,8,9]. In addition, a recent study showed that in these HCV/HIV-co-infected patients, HPgV-1 RNA was associated with a significant reduction in the severity of HCV-related liver disease [13,14,15]. However, several studies have failed to confirm a positive impact of HPgv-1 co-infection on HIV disease [14,16,17]. An explanation for this finding is that the genotypic diversity of HPgV-1 may play a role in modulating disease progression in HIV-1-infected individuals. Furthermore, HPgV-1 genotypes 2 and 5 have been associated with delayed progression of AIDS [7,14,18,19]. Therefore, it is necessary to study HPgV-1 genotypic diversity and examine its clinical impact in larger cohorts with HIV-1 infection. This present study investigates the prevalence and genotypic diversity of HPgV-1 and its impact on disease progression in 1062 HIV-1-positive patients in Yunnan, China.

## 2. Materials and Methods 

### 2.1. Ethical Statements

All subjects gave their informed consent for inclusion before they participated in the study. The study was conducted in accordance with the Declaration of Helsinki, and the protocol was approved by the Ethics Committee of Yunnan Provincial Hospital of Infectious Disease, AIDS Care Center (Approval No. YNACC [2015]-12).

### 2.2. Study Population and Sample Collection

**Figure 1:**
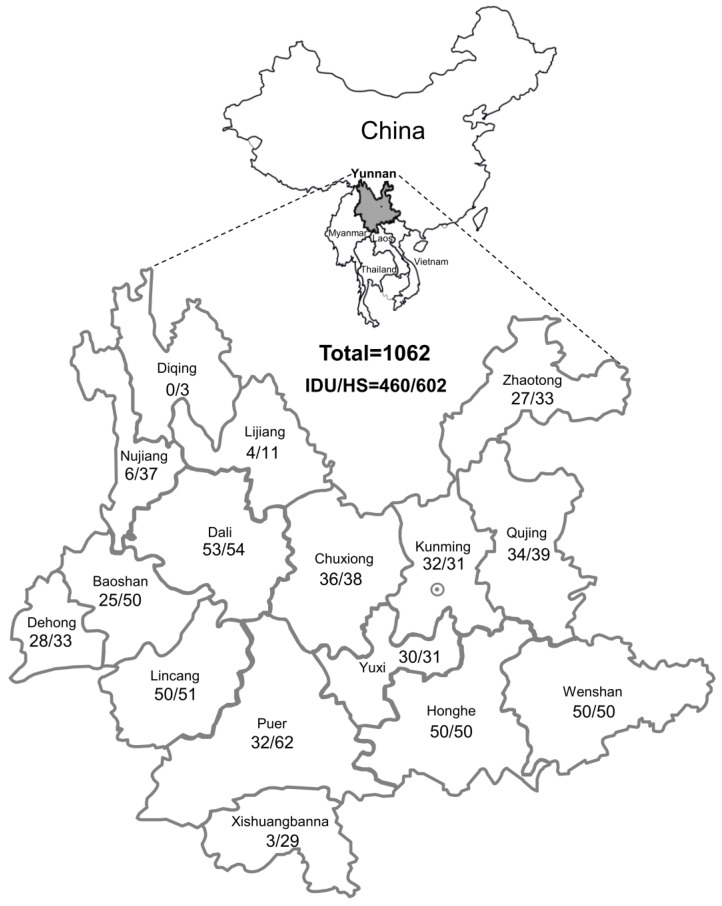
Maps of the study region and geographical distribution of subjects from all sixteen prefectures of the Yunnan province of southwestern China. The Yunnan province of southwestern China is marked in gray. The geographical location of the sixteen regions within this province and the number of samples (IDUs, injecting drug users; HS, heterosexual contact) from each region are shown. This map is modified by the authors according to the free map templates [21] using MapInfo Professional 11.0 (Pitney Bowes Inc., Troy, NY, USA).

In this study, plasma samples were collected from a total of 1062 HIV-positive individuals from Yunnan Provincial Hospital of Infectious Disease, AIDS Care Center (YNACC) between August 2011 and August 2015; these individuals were from each of the 16 prefectures of the Yunnan Province (Figure 1). Blood samples from the individuals were eligible if they were residents of the Yunnan province, self-reported ever having HIV high-risk drug use and sexual behaviors, consent to use patient information in studies of viral epidemics, and able to provide informed consent. Each participant completed a face-to-face interview with trained interviewers to obtain the following information: (1) demographic data, including age, ethnicity, education and marital status etc.; (2) high risk sexual behaviors, including sexual orientation, sexual debut, number of male and/or female sex partners, and experiences of buying or selling sex with other partners in the past 6 months; (3) illicit drug use behaviors, including lifetime history of drug use, age at first injection, and needle sharing. After the interview, an HIV counselor met with the participant for pretest counseling. The interviewer was conducted by trained public health personnel with extensive experience interviewing facial features regarding sexual and drug-use behaviors, and the route of HIV transmission was determined based on analysis of interview data. The samples collected from the participants having multiple risk behaviors and only 18 samples from homosexuals with small sample sizes were excluded from analyses in this study. HIV-1 infection status was determined using an enzyme-linked immunosorbent assay (ELISA) (Abnova, Taibei, Taiwan) and confirmed by western blot assay (MP Biomedicals, Santa Ana, CA, USA). The presence of HCV RNA was detected by RT-nPCR based on the NS5b sequence [20], HCV RNA loads were assessed using the Roche Cobas Amplicor 2.0 assay (Roche, Amsterdam, The Netherlands), and HCV genotype was determined by LIPA 2.0 assay (Siemens Healthcare, Berkeley, CA, USA). Demographic data relating to age, gender, and route of transmission were recorded via self-report questionnaires. The clinical parameters of disease progression, including the alanine aminotransferase (ALT) and aspartate aminotransferase (AST) levels, HIV-1 RNA load, and CD4^+^ T cell counts were determined at sampling time.

### 2.3. RNA Extraction, HPgV-1 Gene Amplification and Sequencing

HPgV-1 RNA was isolated from 200-µL plasma samples using the High Pure Viral RNA Kit according to the procedure described in the manual (Roche, Basel, Switzerland)). Then, the 5’-UTR (U36380:119-497) and *E2* (U36380:950-1844) sequences were amplified by nested PCR; the PCR primers (supplement Table S1) and conditions were as reported in previous study [7,9]. Owing to the high degree of conservation and amplification efficiency of the 5’-UTR, this region was used to evaluate the HPgV-1 infection rate.

The E2 region was used to determine the HPgV-1 genotype, as this sequence could analyze the different genotypes with the same consistency as the complete genome [7,8,9]. The first PCR reaction was performed using One Step reverse transcription PCR (Takara, Dalian, China) and the second using 2 × Taq PCR MasterMix (Tiangen, Beijing, China). The PCR products were firstly detected by agarose gel (1.0%) electrophoresis and visualized under ultraviolet (UV) illumination for the presence of an 895-nucleotide band and then purified by using a DNA purification kit (Tiangen, Beijing, China); subsequently, the purified products were sent to Shenzhen Invitrogen Biotechnology Co., Ltd. (Shenzhen, Guangdong, China) for sequencing by using an ABI 3730XL automated DNA sequencer (Applied Biosystems, Carlsbad, CA, USA).

### 2.4. Sequence Analyses

The sequencing data were initially checked via a NCBI BLAST search [22]. The resulting sequences were edited using BioEdit 7.2.5 software [23]. The reference sequences available in GenBank [24] were downloaded to conduct a comparative analysis of all the HPgV-1 E2 genomic sequences. Multiple alignments of the selected sequences were performed by Clustal Omega [25]. Subsequently, the data generated were processed using the BioEdit 7.1.5 software. Phylogenetic trees were constructed based on the obtained datasets using MEGA version 6.0.6 [26] with maximum-likelihood method using the general time reversible + gamma distribution + invariant sites (GTR + τ + I) model. Bootstrap values were calculated based on 1000 replications of the alignment. Principal coordinate analysis was performed using a principal coordinate analysis (PCOORD). All the HPgV-1 E2 genomic sequences obtained in this study have been deposited in GenBank under accession numbers KX430523-KX430734.

### 2.5. Statistical Analysis

Statistical analyses were conducted using the SPSS 21.0 statistical analysis software package (IBM, Armonk, NY, USA). For descriptive analyses, the means and standard deviations, frequency, and percentage values were reported. The tests of differences between the HPgV-1-infected group and HPgV-1-uninfected group were performed using a *t* test for the difference in means (for age, ATL, AST, CD4^+^ counts, log HIV-1 RNA, and log HCV RNA) and Fisher exact test for gender, HIV-1 risk behavior, and HCV genotypes. All the *p* values below 0.05 were considered to indicate statistical significance.

## 3. Results

### 3.1. Epidemiologic and Demographic Characteristics

Blood samples were collected from a total of 1062 HIV-1-positive individuals from all 16 prefectures of the Yunnan province, from August 2011 to August 2015. Among these, 56.69% (602/1062) had become infected mainly via heterosexual contact and 43.31% (460/1062) by injecting drug user. The epidemiological characteristics of the 1062 subjects included in the present study are summarized in Figure 1. The mean age of the participants was 38.57 ± 10.22 years, and the ratio of males to females was 696:366. The following clinical characteristics were identified: the mean ALT (41.31 ± 47.22 IU/L), the mean AST (44.70 ± 64.59 IU/L), the mean CD4^+^ cell count (301.54 ± 187.58 cells/uL), and HIV-1 RNA (4.05 ± 0.67 log copies/mL). In addition, the gender, ALT, and AST showed highly significant differences among different HIV-1 risk behaviors (IDU vs. heterosexual contact) (Supplement Table S2).

### 3.2. HPgV-1 Infection Status

Out of the 1062 patients with HIV-1 infection, the overall prevalence of HPgV-1 infection was 23.4% (248/1062) via 5’-UTR (378bp) amplification by nested RT-PCR. Among these, 117 (19.4%) of 602 heterosexuals were classified as HPgV-1 RNA-positive and 131 (28.5%) of 460 IDUs tested positive for HPgV-1 RNA. The HPgV-1 positive rate of HIV-1-infected IDUs is significant higher than that of heterosexuals (*p* < 0.05). 

Notably, HPgV-1 infection rates among the HIV-1-infected individuals differed based on age and HIV-1 RNA load as follows: age (in years) 16–30 (29.5%), 31–50 (22.5%), > 50 (16.4%) (*p* = 0.019); HIV-1 RNA load 2.98–4.00 (27.2%), 4.00–5.00 (19.8%), >5 (15.8%) (*p* = 0.005). These results revealed that HPgV-1 infection is associated with increasing age and HIV-1 RNA.

In the sampled prefectures, the HPgV-1 RNA-positive rates among HIV-1-infected individuals were higher than 30%; the rate of HPgV-1-positive individuals was 37.3% in Baoshan, 32.6% in Nujiang, and 30.0% in Kunming. There were no significant differences between these prefectures. However, the HPgV-1 RNA-positive rates among individuals from these prefectures were significantly higher than in those from Xishuangbannan (12.5%), Yuxi (13.1%), and Lijiang (13.3%) (*p* < 0.05) (Supplemental Figure S1A). For the remaining prefectures, the HPgV-1 RNA-positive rate was intermediate (16.2%–27.0%) (Figure S1A). Only three HIV-1-positive subjects were from Diqing; among these, HPgV-1 RNA was not detected.

### 3.3. HPgV-1 Genotypes Distribution

**Figure 2:**
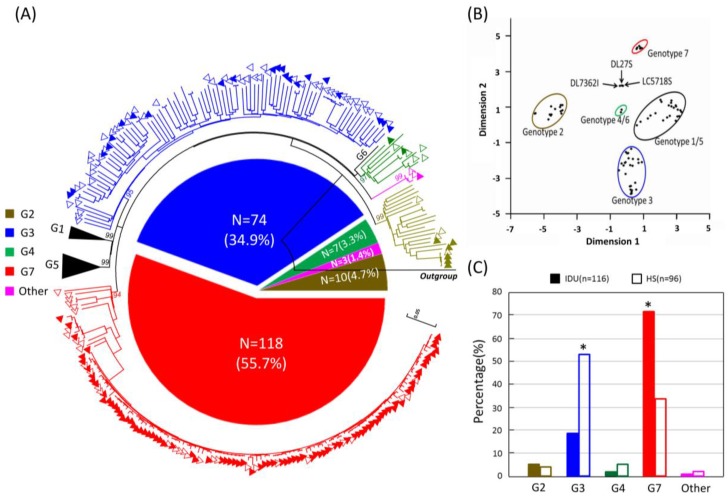
Genotypic analyses of human Pegivirus-1 (HPgV-1) and its distribution between injected drug users and heterosexual individuals in Yunnan, China. (**A**) A circular phylogenetic tree based on the partial *E2* sequences amplified from the 212 human immunodeficiency virus-1 (HIV-1)-infected individuals. The different genotypes are shown in different colors, as indicated on the left of the tree (other means the unclassified group) and the open and closed triangles indicate injecting drug users (IDUs) and heterosexual contact (HS), respectively. The pie chart inside the tree shows the percentages of the various HPgV-1 genotypes; (**B**) Multivariate principal coordinate analysis (PCOORD) of partial *E2* sequences of HPgV-1 (filled circles); (**C**) Comparison of HPgV-1 genotype distributions among IDUs and heterosexuals in Yunnan, China. The distribution significance of genotype 7 and 3 among the IDUs and heterosexuals were presented with asterisks (*p* < 0.05).

Out of a total of 248 HPgV-1 RNA-positive samples, 212 partial *E2* gene fragments were successfully amplified and sequenced with a success rate of 85.5% (212/248). The failure of amplification in the 36 cases was possibly due to low viral load and weak primer specificity. Phylogenetic analyses were performed based on *E2* fragments of HPgV-1. Of these, 118 (55.7%), 74 (34.9%), 10 (4.7%), and 7 (3.3%) sequences were identified as corresponding to genotypes 7, 3, 4 and 2, respectively (Figure 2A). The remaining three strains (DL27S, DL73621 and LC5718S) formed a distinct monophyletic branch with a bootstrap value of 99%, distantly related to all known HPgV-1 genotypes, indicating that these may represent a novel genotype especially when recombination was excluded by bootscanning analyses (no breakpoint was found). Further, the coordinate result obtained by a principal coordinate analysis (PCOOD) indicated that the three strains formed a single group.

Based on the transmission route of HPgV-1, the genotypic distribution among the 116 IDUs was as follows: 85 (73.3%, 85/116), genotype 7; 22 (19.0%, 22/116), genotype 3; 6 (5.2%, 6/116), genotype 2; 2 (1.7%, 2/116), genotype 4; and 1 (0.9%, 1/116), an unclassified group. Among the 96 heterosexuals, genotype 3 was the most common genotype (54.2%, 52/96), followed by genotype 7 (34.4%, 33/96), genotype 4 (5.2%, 5/96), genotype 2 (4.2%, 4/96), and the unclassified group (2.1%, 2/96). Notably, the distribution of genotype 7 among the IDUs (73.3%, 85/116) was significantly higher than among the heterosexuals (34.4%, 33/96) (*p* < 0.05). The percentage of genotype 3 prevalence among heterosexuals (54.2%, 52/96) was significantly higher than in the IDUs (19.0%, 22/116) (*p* < 0.05) (Figure 2C).

In the sampled prefectures, HPgV-1 strains circulating in Baoshan, Dehong, Lincang, Puer, Xishuangbannan and Dali exhibited extremely higher genotypic diversity compared with those in the other prefectures (Figure S1B).

### 3.4. Co-infection of HPgV-1 with HIV/HCV and Its Clinical Effect

**Figure 3:**
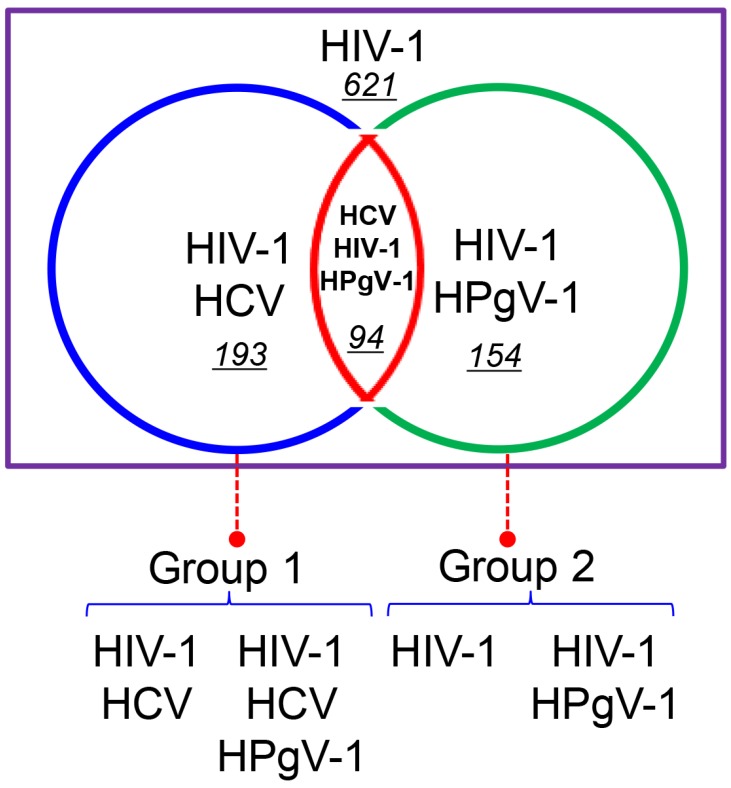
A graphical representation of HIV-1 mono-, HIV-1/Hepatitis C Virus (HCV) co-, and HIV-1/HCV/HPgV-1 triple-infection cases in our cohort in Yunnan, China. 621: the numbers of HIV-1 mono-infection, 193: HIV-1 and HCV co-infection, 154: HIV-1 and HPgv-1 co-infection, and 94: HIV-1, HCV and HPgv-1 triple-infection. Different groups are shown in different colors, as indicated at the bottom of the graphic.

Out of the 1062 HIV-1-infected patients, HCV RNA was detected in plasma samples from 287 (27.02%, 287/1062) subjects using RT-nPCR based on the HCV NS5B region. In combination with the results for HPgV-1 detection, 621 (58.47%, 621/1062) subjects were found to be infected with HIV-1 alone, 193 (18.17%, 193/1062) were co-infected with HIV-1 and HCV, 154 (14.50%, 154/1062) were co-infected with HIV-1 and HPgV-1, and 94 (8.85%, 94/1062) with HIV-1, HCV, and HPgV-1. Taken together, the individuals were further grouped into two comparative groups; group 1 of HIV/HCV co-infected subjects with or without HPgV-1 infection and group 2 of HIV-infected subjects with or without HPgV-1 infection (Figure 3).

Clinical characteristics for patients with and without HPgV-1 infection in the two groups above are summarized in Table 1. The mean (Standard deviation, SD) HIV-1 viral load of HPgV-1-infected patients was lower than patients negative for HPgV-1 (Group 1: log = 3.88 ± 0.47 vs. 4.02 ± 0.61, *p* = 0.040; Group 2: 3.93 ± 0.52 vs. 4.04 ± 0.69, *p* = 0.048, respectively) and the mean (SD) T CD4^+^ lymphocyte cell counts was higher than patients negative for HPgV-1 (Group 1: cells/µL = 322 ± 193 vs. 280 ± 148, *p* = 0.046; Group 2: 322 ± 184 vs. 288 ± 154, *p* = 0.048, respectively). In contrast, there was no significant difference in terms of mean (SD) gender, ALT, AST, transmission route, HCV viral load, and HCV genotype (*p* > 0.05) between the HPgV-1-infected and uninfected patients. 

Interestingly, in the two groups (only the most common genotype of 3 and 7 were compared and genotype 2 and 4 were not included for lacking enough number of individuals), HPgV-1 infection with genotype 7 was significantly associated with lower HIV-1 viral load (Group 1: log = 3.85 ± 0.49 vs. 4.02 ± 0.61, *p* = 0.042 ; Group 2: 3.83 ± 0.64 vs. 4.04 ± 0.69, *p* = 0.038, respectively) and higher CD4^+^ cell counts (Group 1: cells/µL = 332 ± 204 vs. 280 ± 148, *p* = 0.037; Group 2: 341 ± 164 vs. 288 ± 154, *p* = 0.015, respectively) compared with the HPgV-1 negative group (*p* < 0.05) (Table 2). These findings suggest that infection with HPgV-1 genotype 7 may be associated with slower disease progression in HIV-1-positive individuals. In addition, when HPgV-1 genotype 7 compared with genotype 3, there was no significant difference showed.

## 4. Discussion

In the present study, we evaluated the frequency, genotypic distribution, and clinical impact of HPgV-1 infection on HIV-1 disease progression in 1062 HIV-1-infected individuals in Yunnan, China. The overall positive detection rate of HPgV-1 RNA was 23.4% in our cohort (28.5% in IDUs and 19.4% in heterosexuals), which is in accordance with other previous reports stating that HPgV-1 RNA prevalence is about 17%–41% among HIV-positive individuals [19]. Our data indicated that the frequency of HPgV-1 infection in IDUs was significantly higher than in heterosexuals, implying that intravenous injection may represent a more efficient transmission route of HPgV-1 than heterosexual contact. The observation agreed with the finding that intravenous drug use constitutes an important risk behavior for the transmission of blood-borne viruses such as HIV-1, HCV, HBV, and so on [27,28,29].

Moreover, we found that the rate of HPgV-1 infection was significantly higher in younger patients (age: 16-30 years) (*p* = 0.029), and that the frequency of HPgV-1 infection declines with increasing age (*p* = 0.019). Similar trends have been identified in several previous studies [30,31]. This may be attributed to younger individuals being more sexually active and exhibiting higher incidence of drug use than older patients [31,32,33]. Another possible explanation is that E2 antibodies against HPgV-1 reinfection are found more frequently in older people [30,31]. Unfortunately, in the present study, the HPgV-1 E2 antibody levels could not be estimated due to the lack of a commercially available serology assay [34]. In addition, with the increase of HIV-1 RNA viral load, a significant decline in the overall HPgV-1 infection rate was observed, indicating that HPgV-1 infection exhibits an inverse relationship with HIV-1 load. However, we could not draw the conclusion that a negative correlation exists between HPgV-1 and HIV-1 RNA levels. Therefore, further studies are needed to determine the HPgV-1 RNA viral load.

Our results indicate that genotypic diversity and complexity of HPgV-1 circulating in Yunnan, China is high. Multiple genotypes, namely 2, 3, 4, 7, and an unclassified group, were identified in this study; these findings differ from those of previous reports which suggest the predominance of genotype 3 in Beijing [35], and the Hubei province of China [6]. However, our results are consistent with observations in Singapore [36], and Indonesia [37] where high prevalence of HPgV-1 multiple genotypes have been described in patients with HIV-1 infection. These findings may be explained by the special geographic location of Yunnan province. Yunnan is located in southwestern China and borders the opium-producing “Golden Triangle” region composed of Myanmar, Laos, Thailand, and Vietnam [7,38,39]. The transmission of several human viruses is closely associated with illegal drug trafficking [40,41]. Additionally, large numbers of commercial sex workers from Myanmar and Vietnam are active at the Yunnan border [42,43]. These high-risk factors have facilitated the spread of numerous human viruses in Yunnan; for example, HIV-1 subtypes B and C were introduced into Yunnan from Myanmar and India by IDUs [44], and CRF01_AE was identified among commercial sex workers returning from Thailand to Yunnan. In addition [44], such factors have resulted in the transmission of HPgV-1 genotype 4 and 7 in Yunnan. In contrast, genotype 2 predominates in Europe and America [8,9]. A small number of genotype 2 infections detected in Yunnan may have been imported as a result of the frequent exchange of personnel between China and the USA, which has arisen due to the close economic ties between the two countries. Interestingly, we found that HPgV-1 genotype 7 predominated in IDUs, whereas genotype 3 was the most common genotype in heterosexual populations. This pattern and prevalence of HPgV-1 infections may be closely related to the origin of the two kinds of genotypes. HPgV-1 genotype 7 originated in IDUs from Southeast Asia described in our previous study. Genotype 3 may have been derived from China or Japan. To date, little is known about the evolution, origin, and migration of HPgV-1 genotype 3 and 7. Therefore, further molecular epidemiological surveys are needed to monitor the epidemiology and phylogenetic linkages implicated in the HPgV-1 epidemic in Asia.

Several epidemiological studies have indicated a beneficial effect of HPgV-1 viremia on disease progression of HIV-1-infected individuals. HPgV-1 infection has been associated with higher CD4^+^ cell counts and lower HIV-1 loads [7,8,9]. These findings are further supported by increasing evidence for an attenuating effect of HPgV-1 on HIV-1 replication in vitro [45,46,47]. Furthermore, studies of the genetic diversity of HPgV-1 revealed the existence of seven distinct genotypes worldwide; these genotypes present clear differences in regional distribution [8,9]. Interestingly, several reports have suggested that genotypes 2 and 5 are associated with delayed AIDS progression to a greater extent than the other genotypes [7,14,18,19]. Another hypothesis suggests that HPgV-1 may influence the progression of HIV-1 disease; this is the subject of numerous epidemiological surveys [8,9]. However, similar results have been rarely found in cohorts of HIV-1- and/or HCV-positive patients [8,14,16]. This may be attributed to the small number of subjects investigated in these studies, which results in lower accuracy of statistical analyses. In the present study, an investigation of a large cohort of HIV-1-infected patients (1062 subjects) suggested that the recently identified genotype 7 may be more strongly associated with slow HIV-1 disease progression than negative, supporting the hypothesis outlined above.

Our study lacked a long-term follow-up epidemiological survey aimed at HPgV-1 strains with different genotypes. Another limitation of the study is that some demographic and clinical variables, such as race, HPgV-1 E2 antibody levels, HPgV-1 RNA viral load, HIV-1 subtype, and treatment regimen, were not evaluated.

## 5. Conclusions

In summary, our data suggest that HPgV-1 infection among HIV-1-infected populations is extremely common; the HPgV-1 epidemic in Yunnan was characterized by infection with multiple genotypes. We additionally found that HPgV-1 genotype 7 may be associated with slower disease progression in HIV-1-infected individuals. These present findings provide novel insights into the genotypic diversity of HPgV-1 in co-infected HIV-1-positive individuals and are potentially useful for the development of HPgV-1-based bio-therapies for AIDS.

## Figures and Tables

**Table 1:** Differences between HPgV-1-infected and HPgV-1-uninfected patients among HIV-1-infected individuals with or without HCV.

Variable	Group 1: HIV-1/HCV (*n* = 287)			Group 2: HIV-1 (*n* = 775)		
	HPgV-1-Infected	HPgV-1-Uninfected	*p*	HPgV-1-Infected	HPgV-1-Uninfected	*p*
Patients, *n* (%)	94 (32.75)	193 (62.25)	N/A	154 (19.87)	621 (80.13)	N/A
Gender, male:female ^2^	79:15	150:43	0.273	86:71	386:240	0.145
Mean (SD) age, y ^1^	37.99 (5.80)	38.33 (6.05)	0.639	36.90 (11.5)	39.14 (11.34)	0.029
Mean (SD) ALT, IU/L ^1^	51.45 (37.42)	55.48 (43.29)	0.227	31.15 (26.71)	33.03 (29.94)	0.834
Mean (SD) AST, IU/L ^1^	55.26 (36.20)	56.22 (40.03)	0.843	34.46 (23.33)	35.06 (26.24)	0.237
Mean (SD) CD4^+^ count, cells/Ul ^1^	322 (193)	280 (148)	0.046	322 (184)	288.5 (154)	0.048
Mean (SD) HIV-1 RNA log copies/mL ^1^	3.88 (0.47)	4.02 (0.61)	0.040	3.93 (0.52)	4.04 (0.69)	0.048
HIV-1 transmission route no. (%) ^2^			0.530			0.278
Injection drug users	78 (82.98)	154 (79.79)		51 (33.12)	181 (28.91)	N/A
Heterosexual	16 (17.02)	39 (20.21)		103 (66.88)	445 (71.09)	N/A
Mean (SD) HCV RNA logcopies/mL ^1^	4.98 (0.21)	5.12 (0.31)	0.378	N/A	N/A	N/A
HCV genotype no. (%) ^2^			0.563			N/A
1	17 (18.09)	40 (20.73)		N/A	N/A	
2	0 (0.00)	2 (1.04)		N/A	N/A	
3	61 (64.89)	112 (58.03)		N/A	N/A	
6	16 (17.02)	39 (20.20)		N/A	N/A	

N/A, not applicable; *n*, number; y, years; SD, standard deviation; ALT, Alanine aminotransferase; AST, Aspartate transaminase; ^1^ *t* test; ^2^ Fisher exact test.

**Table 2:** Differences between HPgV-1-infected and HPgV-1-uninfected patients among HIV-1-infected individuals with or without HCV.

Variable	HPgV-1-Uninfected	HPgV-1-Infected				
		G3	P1	G7	P2	P3
Group 1: HCV+, n (%)	193 (62.25)	11 (3.83)	N/A	66 (23.00)	N/A	N/A
Gender, male:female ^2^	150:43	8:3	0.714	57:9	0.156	0.363
Mean (SD) age, y^1^	38.33 (6.05)	37.91 (7.35)	0.822	38.11 (5.33)	0.783	0.915
Mean (SD) ALT, IU/L ^1^	55.48 (43.29)	55.48 (43.29)	0.703	50.93 (38.8)	0.463	0.968
Mean (SD) AST, IU/L ^1^	56.22 (40.03)	50.62 (22.56)	0.647	54.61 (36.25)	0.781	0.726
Mean (SD) CD4+ count, cells/uL ^1^	280 (148)	352 (199)	0.144	332 (204)	0.037	0.503
Mean (SD) HIV-1 RNA log10 copies/mL ^1^	4.02 (0.61)	3.97 (0.37)	0.827	3.85 (0.49)	0.042	0.481
HIV-1 transmission route IDUs:HS ^2^	154:39	10:1	0.696	56:10	0.467	0.999
HCV genotype no. (%) ^2^			0.757		0.535	0.753
1	40 (20.73)	2 (18.18)		10 (15.15)		
2	2 (1.04)	0 (0.00)		0(0.00)		
3	112 (58.03)	8 (72.73)		44 (66.67)		
6	39 (20.20)	1 (9.09)		12 (18.18)		
Group 2: HCV-, n (%)	621 (84.37)	63 (8.56)	N/A	52 (7.07)	N/A	N/A
Gender, male:female ^2^	386:240	33:30	0.999	31:21	0.769	0.457
Mean (SD) age, yrs ^1^	39.14 (11.34)	34.73 (12.04)	0.004	38.27 (11.60)	0.596	0.113
Mean (SD) ALT ^1^	33.03 (29.94)	34.5 (4.04)	0.248	33.71 (28.37)	0.879	0.324
Mean (SD) AST ^1^	35.06 (26.24)	34.42 (25.90)	0.855	34.57 (19.62)	0.899	0.896
Mean (SD) CD4^+^ count, cells/uL ^1^	288.5 (154)	310 (195)	0.366	341 (164)	0.015	0.387
Mean (SD) HIV-1 RNA log10 copies/mL ^1^	4.04 (0.69)	3.97 (0.57)	0.258	3.83 (0.64)	0.038	0.349
HIV-1 transmission route IDUs:HS ^2^	181:445	16:47	0.661	24:28	0.012	0.030

P1, HPgV-1 G3 vs. HPgV-1-uninfected; P2, HPgV-1 G7 vs. HPgV-1-uninfected; P3, HPgV-1 G3 vs. HPgV-1 G7; ^1^ *t* test; ^2^ Fisher exact test.

## Supplementary Materials

The following are available online at www.mdpi.com/1999-4915/9/2/28/s1, Table S1: Oligonucleotide primer sequences used to amplify HPgV-1, Table S2: The distribution of HPgV-1 infection rates and genotypes for each prefecture in Yunnan. Figure S1: Demographic and clinical characteristics among HIV-1-infected population.
