# Supplementary material for: Prevalence and Clinical Impact of Human Pegivirus-1 Infection in HIV-1-Infected Individuals in Yunnan, China
Source: Viruses. 2017 Feb 15;9(2):28. doi: 10.3390/v9020028 (PMC5332947; doi:10.3390/v9020028)
Supplement: Supplementary file 1 [file viruses-09-00028-s001.pdf]

Supplementary Materials: Prevalence and Clinical Impact of Human Pegivirus-1 Infection in HIV-1-Infected Individuals in Yunnan, China

Zhijiang Miao, Li Gao, Yindi Song, Ming Yang, Mi Zhang, Jincheng Lou, Yue Zhao, Xicheng Wang, Yue Feng, Xingqi Dong and Xueshan Xia

Table S1. Oligonucleotide primer sequences used to amplify HPgV-1.

Name	Position [a]	RT-PCR Products Size (bp)	HPgV-1 primer sequence [b]	
			F(Forward)	R(Reverse)
GUTRF/R-01	114–515	401	5-GGTTGGTAGGTCGTAAATCCCG-3	5-AATGCCACCCGCCCTCA-3
GUTRF/R-02	119–497	379	5-GTAGGTCGTAAATCCCGGTCA-3	5-CGAAGGATTCTTGGGCTACC-3
GE2F/R-01	940–2216	1276	5-RGTGGGRRAGTGAGTTTTGGAGAT-3	5-GCCTCHGCCAGCTTCATCAGRTA-3
GE2F/R-02	950–1844	894	5-TGAGTTTTGGAGATGGACTGAGCAG-3	5-CGGATYTTGGTCATGGYGTAGGA-3

GUTR, GB virus C untranslated region; GE2, GB virus C envelope protein 2; [a] Nucleotide positions are numbered as for U336380; [b] Mixed base code Y was used for the mixture of C and T; W for A and T; R for A and G; H for A, T and C; V for G, A and C; D for G, A and T.

Table S2. Demographic and clinical characteristics among HIV-1 infected population.

Variable	HIV-1 Infected	HIV-1 Risk Behavior		
		Injection Drug Use	Heterosexual Contact	P
Patients, n (%)	1062	460 (43.31)	602 (56.69)	N/A
Gender, male:female [2]	696:366	387:73	309:293	<0.0001
Mean (SD) age, y [1]	38.57 (10.22)	38.20 (5.82)	38.85 (12.60)	0.265
Mean (SD) ALT, IU/L [1]	41.31 (47.22)	49.16 (41.63)	31.06 (28.20)	<0.0001
Mean (SD) AST, IU/L [1]	44.70 (64.59)	51.70 (35.20)	32.12 (24.70)	<0.0001
Mean (SD) $CD4^+$ count, cells/uL [1]	301.54 (187.58)	299.02 (169.34)	305.58 (190.23)	0.564
Mean (SD) HIV-1 RNA log10 copies/mL [1]	4.05 (0.67)	4.03 (0.63)	3.96 (0.64)	0.093

AST, aspartate aminotransferase; ALT, alanine aminotransferase; SD, Standard Deviation; N/A, not applicable; n, number; y, years; [1] *t* test; [2] Fisher exact test.

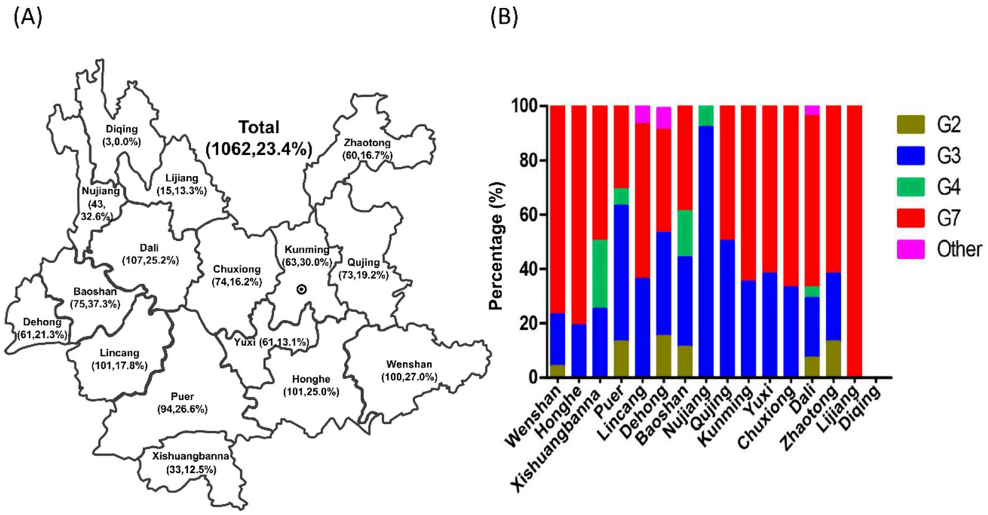

Figure S1. Demographic and clinical characteristics among HIV-1 infected population.

© 2017 by the authors. Submitted for possible open access publication under the terms and conditions of the Creative Commons Attribution (CC BY) license (http://creativecommons.org/licenses/by/4.0/).
